# Circulating concentrations of interleukin (IL)-17 in patients with multiple sclerosis: Evaluation of the effects of gender, treatment, disease patterns and IL-23 receptor gene polymorphisms

**Published:** 2017-01-05

**Authors:** Seyed Ali Ghaffari, Maryam Nemati, Hossain Hajghani, Hossainali Ebrahimi, Abdolkarim Sheikhi, Abdollah Jafarzadeh

**Affiliations:** 1 Neurology Research Center, Department of Neurology, Kerman University of Medical Sciences, Kerman, Iran; 2 Department of Laboratory Sciences, School of Paramedicine, Kerman University of Medical Sciences, Kerman, Iran; 3 Neurology Research Center, Department of Neurology, Kerman University of Medical Sciences, Kerman, Iran; 4 Department of Immunology, School of Medicine, Dezful University of Medical Sciences, Dezful, Iran; 5 Molecular Medicine Research Center, Department of Immunology, School of Medicine, Rafsanjan University of Medical Sciences, Rafsanjan, Iran; 6 Department of Immunology, School of Medicine, Kerman University of Medical Sciences, Kerman, Iran

**Keywords:** Multiple Sclerosis, Interleukin-17, Gender, Treatment, Interleukin-23 Receptor, Gene Polymorphisms

## Abstract

**Background:** Interleukin (IL)-17/IL-23 axis performs a prominent role in the pathogenesis of several autoimmune disorders. This study aimed to investigate the concentrations of IL-17 in patients with multiple sclerosis (MS) and its relationship with gender, medication, disease forms and single nucleotide polymorphisms (SNP) in IL-23R gene, including rs11209026 and rs1004819.

**Methods:** The blood specimens were obtained from 135 healthy individuals and 135 MS patients. The patients exhibited relapsing-remitting (RRMS; n = 65), primary progressive (PPMS; n = 19), secondary progressive (SPMS; n = 35) or progressive relapsing (PRMS; n = 14) MS. The DNA was analyzed for SNPs using polymerase chain reaction-restriction fragment length polymorphism (PCR-RFLP) and IL-17 concentrations were measured by enzyme-linked immunosorbent assay (ELISA).

**Results:** We have observed elevated serum IL-17 concentrations in MS patients compared with healthy individuals (P < 0.001). The men with MS had higher IL-17 concentrations than women patients (P < 0.050). Untreated patients had significantly higher IL-17 concentrations than healthy individuals and treated patients (P < 0.001 and P < 0.010, respectively). The IL-17 concentrations were significantly decreased in patients treated with interferon-β (IFN-β), methylprednisolone or both drugs as compared with untreated MS patients (P < 0.050, P < 0.020 and P < 0.050, respectively). The IL-17 concentrations were also significantly higher in patients with RRMS and PRMS compared with healthy individuals (P < 0.005 and P < 0.010, respectively). The genetic variations at SNPs rs11209026 and rs1004819 were not significantly different between healthy individuals and patients. The IL-17 concentrations were not influenced by genetic variations at investigated SNPs.

**Conclusion:** These results indicated higher levels of IL-17 in MS patients that may be influenced by disease patterns, medication and gender. No association was observed between investigated SNPs and MS.

## Introduction

Multiple sclerosis (MS) is a chronic autoimmune-mediated disease of the central nervous system (CNS) that leads to neuronal demyelination and axonal degeneration.^1^ The clinical courses of MS are defined as relapsing-remitting (RRMS), primary progressive (PPMS), secondary progressive (SPMS) or progressive relapsing (PRMS).^1^ The lymphoid cells, in particular, CD4^+^ T-helper (Th) lymphocytes play a fundamental role in the development of MS and its animal model named experimental autoimmune encephalomyelitis (EAE).^2^ Functionally, separate Th lymphocytes are differentiated from naïve T cells after antigenic stimulation including Th1, Th2, Th17 or regulatory T (Treg) lymphocytes.^2^ Both interferon (IFN)-γ-producing Th1 cells and interleukin (IL)-17-producing Th17 cells are accountable for demyelination in MS and EAE diseases.^2^^,^^3^ In contrast, the Treg cells may confer protection against diseases while the Th2 role remains obscure.^2^^,^^4^ Elevated concentrations of a Th17 cell-associated chemokine (CCL20) and diminished levels of a Th2/Treg cell-related chemokine (CCL22) were indicated in patients with MS.^5^^,^^6^

Th17 cells are characterized by the production of a large number of pro-inflammatory cytokines which include mainly IL-17A (also called IL-17), IL-17F, tumor necrosis factor-α (TNF-α), IL-21, IL-22, CCL20 and granulocyte monocyte-colony stimulating factor (GM-CSF).^3^^,^^7^ IL-17 can influence various cell types such as epithelial cells, endothelial cells, fibroblasts, myeloid cells and synoviocytes.^8^ IL-17 elicits the release of different pro-inflammatory mediators such as CXCL1, CXCL6, CXCL8 IL-1b, IL-6, TNF-α, GM-CSF, macrophage inflammatory protein-2 (MIP-2), monocyte chemoattractant protein-1 (MCP-1) and granulocyte colony-stimulating factor (G-CSF).^3^^,^^8^ IL-17 also acts as a powerful inducer of neutrophil aggregation into the inflammatory tissues.^3^ Th17 cells perform an essential function in the development of a number of autoimmune disorders (such as MS, inflammatory bowel disease, rheumatoid arthritis, systemic lupus erythematosus and psoriasis) and allergy and asthma.^9^

IL-23 is a heterodimer cytokine that is composed of two polypeptide including P19 subunit (specific for IL-23) and P40 subunit (shared with IL-12).^10^ The IL-23 receptor is also a heterodimeric molecule and composed of IL-23R and IL-12Rβ1 which bind to P19 and P40 subunits, respectively.^10^ The main producers of IL-23 are dendritic cells and macrophages and its function is to increase the full activation and maintenance of Th17 lymphocytes.^10^ The antigenic stimulation in presence of both IL-6 and transforming growth factor-β (TGF-β) induces the early differentiation of naive CD4^+^ T cells to Th17 lymhocytes.^11^ However, subsequent interaction with IL-23 is needed for reinforcement and pathogenic activities of Th17 lymhocytes. Indeed, TGF-β and IL-6-induced Th17 lymphocytes are less pathogenic and more exposure to IL-23 is needed for development of inflammatory Th17 lymphocytes.^10^^,^^12^ The relationship between IL-23/IL-17 axis and a number of autoimmune and inflammatory disorders including systemic lupus erythematosus,^13^ spondyloarthritis,^14^ psoriatic arthritis,^15^ Graves' disease,^16^ Crohn's disease,^17^ and EAE and MS^18^ has been reported. 

The IL-23R gene is located on the chromosome 1p31.3 that encodes the receptor for IL-23.^19^ The association of the IL-23R gene polymorphisms with several inflammatory and autoimmune diseases such as ankylosing spondylitis,^20^ psoriatic arthritis,^21^ allergic rhinitis,^22^ rheumatoid arthritis,^23^ and systemic lupus erythematosus^24^ has been reported. Recently, in one study the associations of several single nucleotide polymorphisms (SNPs) within IL-23R gene (rs2201841, rs10889677 and rs7517847) with MS have been investigated in a Chinese population and showed no association between these SNPs and MS disease.^25^

The SNP rs11209026 (Arg381Gln) leads to replacement of Arg with Gln within the IL23R-binding domain for the JAK2 kinase, and therefore may change the downstream signaling pathways and responses to IL-23.^19^^,^^26^ The much less common glutamine allele seems to protect against some autoimmune diseases such as inflammatory bowel disease, psoriasis and ankylosing spondylitis.^19^^,^^27^ The association of SNP rs11209026 with a number of immune-related diseases such as inflammatory bowel disease,^28^ psoriasis,^29^ Crohn's disease^30^ has also been reported. The SNP rs1004819 is located in the intronic region of the IL-23R gene and may exert its influence by regulating the splicing of IL-23R mRNA.^19^ However, a considerable association between SNP rs1004819 with ulcerative colitis^31^ and ankylosing spondylitis^19^ has also been indicated in other investigations. The aforementioned SNPs may influence the expression and function of the IL-23R; however, their relationship with MS have not been investigated in Iranian populations, yet. 

Although a number of studies have evaluated the contribution of IL-17 in the development of MS, its concentrations have not been assessed adequately in MS patients with respect to gender, medication, forms of MS and IL-23R gene polymorphisms,. Accordingly, this study was conducted to assess the concentrations of IL-17 in patients with MS and its relationship with gender, medication, disease patterns and SNPs in IL-23R gene, such as rs11209026 and rs1004819.

## Materials and Methods

A total of 135 MS patients (29 men and 106 women) referring to the Shephah Hospital of Kerman (a city placed in southeast Iran) were enrolled into the investigation. Sixty five patients presented with RRMS, 19 presented with PPMS, 14 had PRMS and 35 presented with SPMS. The diagnosis of MS was verified by expert neurologists based on the reliable diagnostic findings including clinical and paraclinical examinations [magnetic resonance imaging, presence of oligoclonal bands in cerebrospinal fluid (CSF) and evoked potentials according to the McDonald’s criteria.^32^ The MS patients were classified as newly diagnosed (untreated) patients (n = 47) who were not treated with any drug, and previously diagnosed (treated) patients (n = 88) who were treated with methylprednisolone (intravenously by a dose of 1000 mg/day for 3 to 5 days after acute MS attack), IFN-β [Avonex (Biogen Idec Co, USA; 30 μg intramuscularly, one time weekly), CinnoVex (CinnaGen Co, Iran; 30 μg intramuscularly, once time weekly) or Rebif (Merck Biopharma Co, Germany; 44 μg subcutaneously, three times weekly)] for at least 3 months. A number of MS patients initially received IFN-β (during silent phase of disease) and later methylprednisolone after having an acute attack of MS. 

Also 135 healthy individuals including 35 men and 100 women were enrolled into the investigation as a control group. The healthy individuals were selected among blood donors referring to the Blood Transfusion Organization of Kerman and were interviewed regarding CNS disorders and none of them had CNS or other related disorders. All control individuals were basically healthy, with no acute or chronic sickness. Indeed individuals with illness (such as any suspected immunological disorders, history of recurrent infections, asthma, allergy and atopic diseases), cigarette smoking and those taking any medication were excluded from the study. The other exclusion criteria were malignancy, surgery and major trauma within 6 months prior to blood collection. 

The Ethical Committee of Kerman University of Medical Sciences evaluated and approved the investigation. In addition, the participants were enrolled into the study on their own wish and informed written consent was also obtained from all of them. A peripheral blood specimen (2-4 ml) was taken from all participants and the sera were separated and stored at -70 °C until analysis. 

***DNA extraction and genotype analysis*** *:* Genomic DNA was separated from peripheral white blood cells by salting out technique as previously described by Miller, et al.^33^ The purity and the quantity of DNA specimens were determined by the detection of the optical density at 260 and 280 nm wavelengths using a spectrophotometry system (Ependorf, Germany). DNA specimens were kept at -20 °C until testing. The genetic variations at SNPs rs11209026 and rs1004819 in IL-23R gene was determined by utilization of polymerase chain reaction-restriction length polymorphism (PCR–RFLP) technique. 

The PCR reaction mixture was formed by adding following reagents to a 0.2-ml microcentrifuge tube on ice: 2.5 μl of Taq DNA polymerase buffer (10×), 0.5 μl of MgCl2 (stock concentration 1.5 mM), 0.5 μl of each dNTP [dATP, dCTP, dGTP, and dTTP (stock concentration of 10 mM)], 1 μl of each primer, 1 μl of prepared DNA, and sterile double-distilled water to a final volume of 25 μl.

For SNP rs11209026, the sequences of primers were 5’-AGTCACTCTGTGGCCTAAAGTAAAG-3’ for the forward primer and 5’-AGATTTTTCTAGTAAACAACTGAAATGA-3’ for the reverse primer. Amplification was done with the following thermocycler program: 

One early phase of 94 °C for 5 minutes, followed by 35 cycles of 95 °C for 30 seconds, 61 °C for 1 minute and 72 °C for 90 seconds. The amplified PCR product of IL-23R gene covers the SNP rs11209026 with a molecular size of 350 bp. The Hpy1 88I (Fermentase, Finland) restriction enzyme had been used for digestion of the PCR product. The digested products were electrophoresed on a 2.5% agarose gel after adding 4 μl of loading buffer (Cinnagen, Iran) and studied on Chemi-Doc model XRS (Bio-Rad, USA) after staining with ethidium bromide. As reported previously,^19^ in the situation of heterozygotic form (AG), four different fragments with 285, 250, 65 and 35 bp are produced. In the GG homozygotic form, three fragments (250, 65 and 35 bp) and in the AA homozygotic form, two fragment (85 and 65 bp) are formed. 

For SNP rs1004819 the sequences of primers were 5’-GCATTCTAG GACCGTTTTGG-3’ for the forward primer and 5’-ATCTGGTGGAAATATGTGAAACCTAA-3’ for the reverse primer. The amplification was performed with the following thermocycler program: 

One primary phase of 95 °C for 5 minutes, followed by 35 cycles of 95 °C for 30 seconds, 61 °C for 1 minute and 72 °C for 90 seconds. The amplified PCR product of IL-23R gene covers the SNP rs1004819 with a molecular size of 270 bp. The TaaI (Fermentase, Finland) restriction enzyme had been used for digestion of the PCR product. The digested products were electrophoresed as mentioned above. As reported previously,^34^ in the situation of homozygotic form (GG), three different fragments with 185, 71 and 13 bp are produced. In the AA homozygotic form, two fragments (185 and 65 bp) and in the GA heterozygotic form four fragment (257, 185, 71 and 13 bp) are observed. 

***Cytokine assay:*** The serum IL-17A concentrations were measured in duplicate by commercial enzyme-linked immunosorbent assay (ELISA) kits (Mabtech, Sweden) based on the manufacturer’s instructions. The sensitivity of the kits was less than 4 pg/ml and the intra-assay variation was < 5%. 

The differences in variables were analyzed using parametric statistical tests (including ANOVA and Student’s t-tests for normal distribution of data) and non-parametric statistical tests (including Kruskal-Wallis and Mann-Whitney U tests for non-normal distribution of data) as appropriate. A P-value of less than 0.05 was considered significant. The data were analyzed using SPSS (version 15, SPSS Inc., Chicago, IL, USA).

## Results

The mean ages for MS patients and healthy individuals were 35.70 ± 7.90 years and 36.50 ± 7.79 years, respectively (P = 0.410). The gender distribution was 106 women (78.5%) and 29 men (21.5%) in MS patients, and 100 women (74.1%) and 35 men (25.9%) in healthy individuals (P = 0.470). 

**Figure 1:**
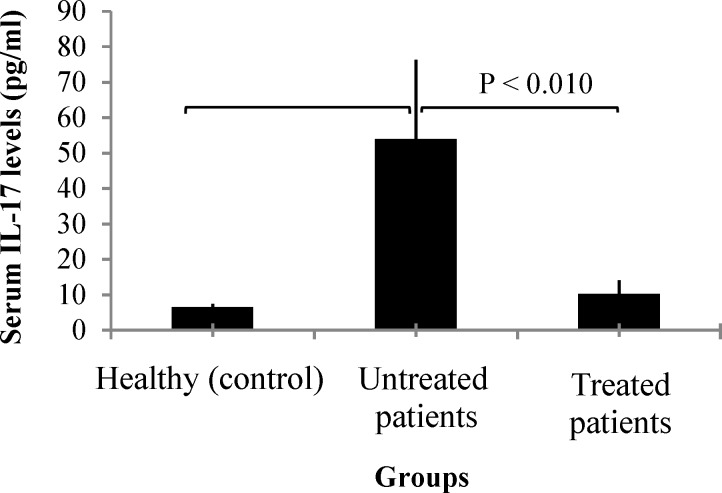
Comparison of the serum interleukin-17 (IL-17) levels between healthy control group, untreated and treated multiple sclerosis (MS) patients

***Serum IL-17 concentrations in MS patients and healthy individuals:*** We have observed elevated serum IL-17 concentrations in MS patients compared with healthy individuals (P < 0.001) (Table 1). The men with MS had higher IL-17 concentrations compared with women patients (P < 0.050). In healthy individuals, no significant difference was observed between men and women regarding the IL-17 concentrations. In both men and women patients the IL-17 concentrations were significantly higher than the healthy individuals with same gender (P < 0.002 and P < 0.010, respectively) (Table 1) (Figure 1).

**Table 1:** The serum interleukin-17 (IL-17) concentrations in multiple sclerosis (MS) and healthy groups according to gender

**Groups**	**Gender**	**n**	**IL-17 levels** † ** (mean ± SD)**	**P**
MS patients	Male	29	56.30 ± 28.60	0.050*
	Female	106	17.00 ± 7.03	0.001**
	Total	135	25.50 ± 8.31	-
Healthy (control)	Male	35	4.50 ± 0.80	0.260*
	Female	100	7.11 ± 1.35	
	Total	135	6.43 ± 1.03	

IL: Interleukin; MS: Multiple sclerosis; SD: Standard deviation

† The serum levels of cytokine expressed as pg/ml,

* Represent the difference between men and women in each group,

** Represent the difference between MS and healthy groups

**Table 2:** The serum interleukin-17 (IL-17) concentrations in newly untreated and treated multiple sclerosis (MS) patients according to their gender

**Groups**	**Sex**	**n**	**IL-17 levels** † ** (mean ± SD)**	**P**
Untreated MS patients	Male	12	121.10 ± 66.00	0.050*
	Female	35	30.90 ± 19.10	0.001**
	Total	47	53.90 ± 22.40	-
Treated MS patients	Male	17	10.70 ± 2.45	0.950*
	Female	71	10.20 ± 4.60	0.240**
	Total	88	10.30 ± 3.74	-
Healthy	Male	35	4.50 ± 0.80	0.260*
	Female	100	7.11 ± 1.35	
	Total	135	6.43 ± 1.02	

IL: Interleukin; MS: Multiple sclerosis; SD: Standard deviation

† The serum levels of cytokine expressed as pg/ml,

* Represents the difference between men and women in each group,

** Represents the difference between indicated and healthy groups

The IL-17 levels in untreated patients was significantly higher than healthy group and treated patients (P < 0.001 and P < 0.010, respectively)

**Figure 2:**
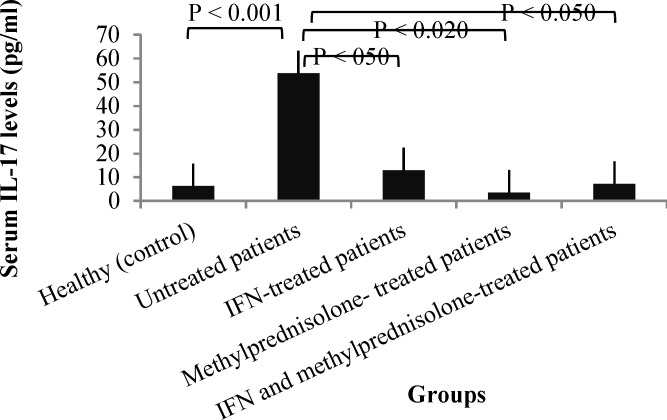
The serum levels of interleukin-17 (IL-17) in multiple sclerosis (MS) patients according to treatment program

***Serum IL-17 concentrations in untreated and treated MS patients:*** The mean serum IL-17 concentrations in newly diagnosed MS patients was significantly higher than healthy individuals and treated MS patients (P < 0.001 and P < 0.010, respectively) (Table 2) (Figure 2). However, the difference of the mean serum IL-17 concentrations in treated MS patients and healthy individuals was not statistically significant (P = 0.240). The newly diagnosed patients had RRMS (n = 20), PPMS (n = 14), PRMS (n = 10) or SPMS (n = 3) patterns of disease. There were no significant differences between serum IL-17 concentrations from newly diagnosed patients with regard to their pattern of disease. 

**Figure 3:**
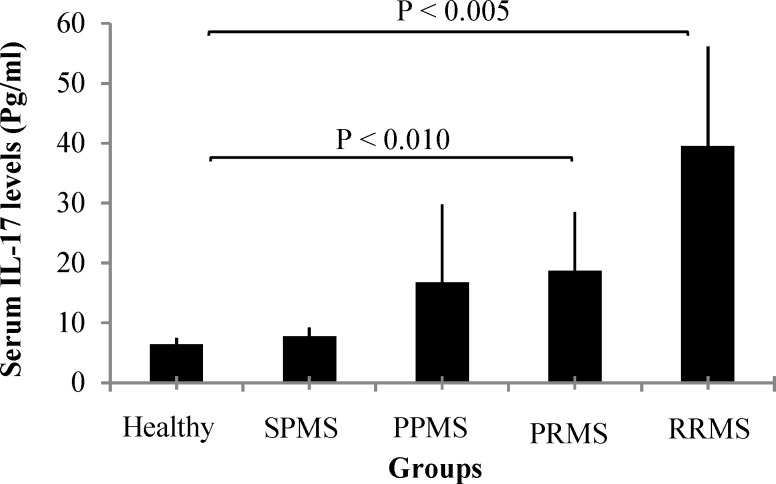
The serum levels of IL-17 in MS patients according to disease patterns

The mean serum IL-17 concentrations in untreated MS men was significantly higher than healthy men or treated men with MS (P < 0.010 and P < 0.050, respectively). Serum IL-17 concentrations in treated men with MS was also significantly higher than healthy men (P < 0.010) (Figure 3).

The mean serum levels of IL-17 in untreated patients was also significantly higher than that in healthy group (P < 0.001). The mean serum levels of IL-17 was significantly lower in patients treated with IFN-β, methylprednisolone or both IFN-β and methylprednisolone as compared to untreated

The mean serum IL-17 concentrations in untreated women with MS was also significantly higher than healthy women (P < 0.050). The difference of the mean serum IL-17 concentrations between untreated-and treated MS women was not significant (P < 0.160). The difference of the serum IL-17 concentrations in untreated women with MS and healthy women was also not significant (P < 0.400). Serum IL-17 concentrations in untreated men with MS was markedly higher than untreated women with MS (P < 0.070). In treated MS patients, no significant difference was observed between men and women regarding the IL-17 concentrations (Table 2). 

***IL-17 concentrations in MS patients according to medication program:*** The serum IL-17 concentrations in MS patients, according to their medication program are demonstrated in table 3. 

**Table 3:** The serum interleukin-17 (IL-17) concentrations in multiple sclerosis (MS) patients according to treatment programs

**Groups**	**Treatment**	**n**	**IL-17 levels** * ** (mean ± SD)**	**P**
MS patients	Interferon	55	12.97 ± 5.92	0.120**
	Methylprednisolone	15	3.65 ± 0.90	
	Methylprednisolone + interferon	10	7.30 ± 3.47	
	No treatment	47	53.90 ± 22.40	-
Healthy control	-	135	6.43 ± 1.02	-

IL: Interleukin; MS: Multiple sclerosis; SD: Standard deviation

* The serum levels of cytokine expressed as pg/ml,

** Represent the difference between MS patients with different treatment

In patients with RRMS and PRMS patterns the mean serum levels of IL-17 was significantly higher than that observed in healthy control group (P < 0.005 and P < 0.01, respectively).

The IL-17 concentrations were significantly decreased in patients treated with IFN-β, methylprednisolone or both drugs as compared with untreated MS patients (P < 0.050, P < 0.020 and P < 0.050, respectively) (Table 4). The differences of the serum IL-17 concentrations between patients treated with methylprednisolone or IFN-β plus methylprednisolone and healthy individuals were not significant (Table 4).

***IL-17 concentrations in MS patients according to disease patterns:*** Statistical analyses indicated that the differences of the serum IL-17 concentrations between MS patients with different disease forms were not significant (Table 5). In patients with RRMS and PRMS patterns, serum IL-17 concentrations were significantly higher than healthy group (P < 0.005 and P < 0.010, respectively) (Table 6). Although, in patients with PPMS serum IL-17 concentrations were higher than healthy individuals but the difference was not significant (P < 0.060). The mean serum IL-17 concentrations were similarly expressed in patients with SPMS and healthy individuals (Tables 5 and 6).

***The relation between SNPs rs11209026 and rs1004819 and MS:*** The genetic variations at SNPs rs11209026 and rs1004819 in the IL-23R gene in the patients with MS and the healthy individuals are demonstrated in tables 7 and 8. There was no deviation from equilibrium of Hardy-Weinberg neither in patients nor in healthy individuals regarding the genotype frequencies. There were no significant differences between MS patients and control subjects regarding the frequencies of genotypes and alleles at SNPs rs11209026 and rs1004819. Moreover, there were no significant differences between patients with RRMS, SPMS, PPMS and PRMS patterns regarding the frequencies of genotypes and alleles at SNPs rs11209026 and rs1004819.

Serum IL-17 concentrations according to the genetic variations at SNPs rs11209026 and rs1004819 in IL-23R gene were demonstrated in table 9 and table 10. No significant differences were observed between individuals with different genotypes or alleles at SNPs rs11209026 and rs1004819 with respect to the serum IL-17 concentrations neither in MS patients nor in healthy individuals.

**Table 4:** Statistical comparison of the serum interleukin-17 (IL-17) concentrations between multiple sclerosis (MS) patients, according to their treatment program

**Treatment program**	**IFN-β**	**Methylprednisolone**	**IFN-β + Methylprednisolone**	**No treatment**
IFN-β	-	0.12^*^	0.41	0.050
Methylprednisolone	0.12	-	0.24	0.020
IFN-β + Methylprednisolone	0.41	0.24	-	0.050
No treatment	0.05	0.02	0.05	-
Healthy (control)	0.28	0.05	0.81	0.001

IL: Interleukin;

IFN: Interferon

* The symbol represents P-values;

**Table 5:** Serum interleukin-17 (IL-17) concentrations in multiple sclerosis (MS) patients according to disease patterns

**Groups**	**Diseases patterns**	**n**	**IL-17 levels** * ** (mean ± SD)**	**P**
MS patients	RRMS	65	39.50 ± 16.60	0.001**
	SPMS	37	7.73 ± 1.46	
	PPMS	19	16.80 ± 13.00	
	PRMS	14	18.70 ± 9.79	
	Total	135	16.00 ± 4.21	
Healthy (control)	-	135	6.43 ± 1.02	

RRMS: Relapsing-remitting multiple sclerosis; SPMS: Secondary progressive multiple sclerosis; PPMS: Primary progressive multiple sclerosis; PRMS: Progressive relapsing multiple sclerosis; IL: Interleukin; MS: Multiple sclerosis; SD: Standard deviation

* The serum levels of cytokine expressed as pg/ml,

** Represent the difference between all groups (healthy subjects and MS patients with various disease patterns)

**Table 6:** Statistical comparison of serum interleukin-17 (IL-17) concentrations between multiple sclerosis (MS) patients, according to their disease patterns

**Disease patterns**	**RRMS**	**SPMS**	**PPMS**	**PRMS**
RRMS	-	0.15*	0.28	0.28
SPMS	0.150	-	0.34	0.28
PPMS	0.280	0.34	-	0.90
PRMS	0.280	0.28	0.90	-
Healthy (control)	0.005	0.53	0.06	0.01

RRMS: Relapsing-remitting multiple sclerosis; SPMS: Secondary progressive multiple sclerosis; PPMS: Primary progressive multiple sclerosis; PRMS: Progressive relapsing multiple sclerosis

* The symbol represents P-values

## Discussion

Results from our study indicated that increased serum concentrations of IL-17 in MS patients that is consistent with findings reported by other investigators. In both male and female patients the concentrations of IL-17 were significantly higher in comparison with healthy individuals with the same gender. According to these results, increased concentrations of IL-17 might have a role in the pathogenesis of MS in either males or females.

**Table 7:** The frequencies of genotypes and alleles at rs11209026 in interleukin-23 (IL-23R) gene in patients with multiple sclerosis (MS) and healthy control group

**Genotype**	**MS patients ** **[n (%)]**	**Healthy subjects ** **[n (%)]**		**P**
GG	126 (93.3)	124 (91.90)	0.640	
GA	9 (6.70)	11 (8.10)	0.640	
G	261 (96.67)	259 (95.90)	0.640	
A	9 (3.33)	11(4.07)	0.640	

MS: Multiple sclerosis

The autoreactive Th17 lymphocytes have shown to be at high frequencies in the CNS of EAE mice and in the peripheral blood mononuclear cells (PBMCs) of MS patients.^35^^,^^36^

**Table 8:** The frequencies of genotypes and alleles at rs1004819 in IL-23R gene in patients with multiple sclerosis (MS) and healthy control group

**Genotype**	**MS patients ** **[n (%)]**	**Healthy subjects ** **[n (%)]**		**P**
GG	44 (32.6)	36 (26.7)	0.280	
GA	63 (46.7)	73 (54.1)	0.220	
AA	28 (20.7)	26 (19.3)	0.760	
A	119 (44.1)	125 (46.3)	0.220	
G	151 (55.9)	145 (53.7)	0.260	

MS: Multiple sclerosis

**Table 9:** The serum interleukin-17 (IL-17) concentrations according to the genetic variations at rs11209026 in IL-23R gene

**Groups**	**Genotypes**	**n (%)**	**IL-17 levels** ^*^ ** (mean ± SD)**	**P**
MS	GG	126 (93.3)	26.60 ± 8.89	0.590
	GA	9 (6.7)	8.95 ± 4.45	
	G	261 (96.7)	26.00 ± 6.06	0.600
	A	9 (3.3)	8.95 ± 4.45	
Healthy	GG	124 (91.9)	6.54 ± 1.11	0.700
	GA	11 (8.1)	5.15 ± 1.74	
	G	259 (95.9)	6.49 ± 0.75	0.710
	A	11 (4.1)	5.15 ± 1.74	
Total	GG	250 (92.6)	16.70 ± 4.55	0.540
	GA	20 (7.4)	6.86 ± 2.19	
	G	520 (96.3)	16.30 ± 3.09	0.550
	A	20 (3.7)	6.86 ± 2.19	

IL: Interleukin; SD: Standard deviation

**Table 10:** The serum interleukin-17 (IL-17) concentrations according to the genetic variations at rs1004819 in IL-23R gene

**Groups**	**Genotype**	**n (%)**	**IL-17 levels** ^*^ ** (mean ± SD)**	**P**
MS	GG	44 (32.59)	29.11 ± 16.83	0.950
	GA	63 (46.66)	23.40 ± 11.30	
	AA	28 (20.74)	24.38 ± 16.76	
	G	151 (55.92)	25.75 ± 9.65	0.440
	A	119 (44.07)	23.70 ± 1.37	
Healthy	GG	36 (26.66)	4.13 ± 0.73	0.250
	GA	73 (54.04)	6.58 ± 1.35	
	AA	26 (19.25)	9.19 ± 3.60	
	G	145 (53.70)	5.77 ± 0.94	0.450
	A	125 (46.29)	7.27 ± 1.37	
Total	GG	80 (29.62)	17.87 ± 9.32	0.730
	GA	136 (50.37)	14.37 ± 5.31	
	AA	54 (20.00)	17.06 ± 8.84	
	G	296 (109.62)	15.67 ± 4.97	0.790
	A	244 (90.37)	15.14 ± 4.54	

The elevated IL-17 concentrations in the CSF of patients with MS also represent the contribution of this cytokine in the development of disease.^36^ In addition, RORγt (a Th17-specific transcription factor)-deficient mice were more resistant to EAE induction.^2^ Low EAE scores, delayed onset and low histological changes with early disease recovery were also reported in IL-17-deficient mice.^2^ However, the mice treated with the monoclonal antibody against IL-17 were reported to have the ability to develop EAE, even with a decreased disease severity.^37^ In addition to IL-17A, other cytokines including IL-17F, GM-CSF, IL-6, IL-21, IL-22 and TNF-α are produced by Th17 lymphocytes, which can play a prominent role in the development of EAE and MS diseases.^3^^,^^8^

The Th17 lymphocytes may be involved in the establishment of MS and EAE by recruitment of neutrophils into the CNS, induction of the reactive oxygen species (ROS) in CNS endothelial cells, activation of microglia cells to secrete pro-inflammatory mediators, and the induction of astrocytes to release CXC chemokines.^3^ Some Th17 lymhocyte-related cytokines (including TNF-α) elicit the expression of matrix metalloproteinases, which may play a considerable role in rupture of blood-brain barrier (BBB) during MS.^38^

In the current study, the men with MS had significantly more serum IL-17 concentrations compared to the female patients. This difference may attribute largely to the effect of sex hormones. In a number of experimental models of inflammatory diseases, it has been indicated that the frequency of Th17 lymphocytes was higher in male as compared with female gender and this phenomenon has been attributed to the inhibitory effects of estrogen on the Th17 cells differentiation.^39^

The results obtained from our study demonstrated that the patients presenting RRMS and PRMS patterns had significantly higher serum IL-17 concentrations in comparison with healthy individuals. Although serum IL-17 concentrations in patients presenting with PPMS were also markedly higher than healthy individuals, but the difference did not reached to a significant value (P < 0.060). These results represented that IL-17 may contribute at least to the pathogenesis of RRMS and PRMS patterns. Consistent with our results, higher frequency of Th17 lymphocytes were reported in patients presenting with RRMS, SPMS and PPMS.^40^^,^^41^ Serum IL-17 concentrations were similar in patients presenting with SPMS and control individuals. In agreement with our finding, the results reported by Frisullo, et al. also showed no significant difference between patients presenting SPMS and healthy individuals regarding the IL-17 production by their PBMC. Therefore, it has been proposed that the initial phase of MS disease is strongly associated with IL-17.^42^

Based on the present results, the serum concentrations of IL-17 were significantly lower in patients treated with IFN-β and/or methylprednisolone compared with untreated patients. It has been reported that IFN enhances activation-induced apoptosis in Th17 lymphocytes through binding to its receptor that is expressed on these cells.^43^ Moreover, IFN-β down-regulates the expression of RORγt, IL-17A and IL-23R, but up-regulates the expression of IL-10 in the CD4^+^ T lymphocytes.^44^ The diminished expression of IL-27 in the CNS of EAE mice was observed in our previous study.^45^ IFN-β may mediates its therapeutic effects through the induction of IL-27 production, which in turn inhibits Th17 cell-related responses.^46^ The PBMC from non-responder MS patients to IFN-β therapy produced lower IL-27 concentrations than responder patients following in vitro stimulation.^46^

The modulatory effects of methylprednisolone on Th17 lymphocytes were reported in a number of studies. methylprednisolone down-regulates the production of IL-17 by PBMC from asthmatic children or patients with rheumatoid arthritis.^47^^,^^48^ Moreover, the reducing effects of methylprednisolone on the frequency of Th17 lymphocyte were demonstrated in patients with MS disease.^49^ In addition, IFN-β and methylprednisolone may up regulate the expression of IL-35 (a Treg-type cytokine) in MS patients.^50^ Based on the present results, the immunomodulatory effects of IFN-β and/or methylprednisolone may perform in part, via the inhibition of the IL-17 production. 

We found no significant differences for frequencies of genotypes and alleles at SNPs rs11209026 and rs1004819 between patients with MS and healthy individuals. These data represent that investigated SNPs may have no association with MS disease. 

Several mechanisms have been suggested by which polymorphisms can change the function of the receptor. The SNPs may also influence the expression of the IL-23R (e.g. by enhancing mRNA stability), therefore, they reinforce the differentiation of native T cells towards a Th17 cells which results in an increased secretion of other inflammatory cytokines. However, our results represent that MS may develop through the mechanisms independent of SNPs rs11209026 and rs1004819. The results of a number of studies have reported no association between the SNP rs1004819 and immune-related diseases including ankylosing spondylitis,^51^ systemic lupus erythematosus,^52^ inflammatory bowel disease^53^ and rheumatoid arthritis.^23^ It should be also noted that a SNP may be in disequilibrium linkage with other SNPs, unlikely conferring independent influence.

No significant differences were observed between subjects with various genotypes or alleles at SNPs rs11209026 and rs1004819 with respect to serum IL-17 concentrations neither in MS patients nor in healthy individuals. These results represent that the serum concentrations of IL-17 were not influenced by genetic variations at SNPs rs11209026 and rs1004819 in MS patients. It has been reported that the SNP rs11209026 influences serum IL-17 concentrations in patients with rheumatoid arthritis.^26^ Whether SNPs rs11209026 and rs1004819 may affect IL-17 concentrations in CNS or CSF during the development of MS remains to be determined in future studies.

## Conclusion

In conclusion, the results obtained from the current research showed higher IL-17 concentrations in MS patients, especially in patients with RRMS and PRMS patterns. Treatment of MS patients with IFN-β and/or methylprednisolone had reducing effects on the IL-17 levels. The serum levels of IL-17 may also be influenced by the gender of patients. However, there was no association between the investigated SNPs rs11209026 and rs1004819 and MS. Also the serum levels of IL-17 were not influenced by genetic variations at SNPs rs11209026 and rs1004819.

## Notes:

**How to cite this article:** Ghaffari SA, Nemati M, Hajghani H, Ebrahimi H, Sheikhi A, Jafarzadeh A. Circulating concentrations of interleukin (IL)-17 in patients with multiple sclerosis: Evaluation of the effects of gender, treatment, disease patterns and IL-23 receptor gene polymorphisms. Iran J Neurol 2017; 16(1): 15-25.
